# Case Report: Genetic Creutzfeldt–Jakob Disease With a G114V Mutation and One Octapeptide Repeat Deletion as a Mimic of Frontotemporal Dementia

**DOI:** 10.3389/fneur.2022.888309

**Published:** 2022-06-24

**Authors:** Xue Lin, Yichen Xu, Zhen Zhen, Kang Xiao, Xu Chen, Jigang Yang, Hongzhi Guan, Qi Shi, Xiaoping Dong, Jiawei Wang, Yanjun Guo

**Affiliations:** ^1^Department of Neurology, Beijing Tongren Hospital, Capital Medical University, Beijing, China; ^2^Department of Neurology, Beijing Puren Hospital, Beijing, China; ^3^Department of Neurosurgery, Beijing Tiantan Hospital, Capital Medical University, Beijing, China; ^4^Department of Neurology, People's Hospital of Beijing Daxing District, Beijing, China; ^5^State Key Laboratory for Infectious Disease Prevention and Control, Collaborative Innovation Center for Diagnosis and Treatment of Infectious Diseases, National Institute for Viral Disease Control and Prevention, Chinese Center for Disease Control and Prevention, Beijing, China; ^6^Department of Neurosurgery, Beijing Friendship Hospital, Capital Medical University, Beijing, China; ^7^Department of Nuclear Medicine, Beijing Friendship Hospital, Capital Medical University, Beijing, China; ^8^Department of Neurology, Peking Union Medical College Hospital, Chinese Academy of Medical Sciences, Beijing, China

**Keywords:** genetic Creutzfeldt-Jakob disease, prion, PRNP, G114V mutation, one octapeptide repeat deletions

## Abstract

Genetic Creutzfeldt–Jakob disease (gCJD) characterized by mutations in the prion protein (PrP) gene (*PRNP*) contributes to approximately 10–15% of the overall human prion diseases. Here, we report a rare mutation in the *PRNP* gene in a Han-Chinese family. A 36-year-old man initiated with anxiety and depression followed by progressive dementia, cogwheel-like rigidity combined with tremors, and he was diagnosed with frontotemporal lobar dementia in the first 2 years. The disease progression was relatively slow, and the patient developed into akinetic mutism in 4 years. To characterize the disease, following the pedigree studies, neuropsychological examination, neuroimaging studies, real-time quaking-induced conversion (RT-QuIC) examination, and so on were conducted. We eventually identified a rare mutation of G114V combined with one octapeptide repeats deletion (1-ORPD) in the PrP in the patient by DNA sequencing. In addition, the same mutation and deletion were subsequently identified in the patient's mother without any syndromes. His maternal grandmother had a late onset of the disease in her 60s. Given that 1-OPRD has never been reported in human prion disease before, our first report that both G114V mutation and 1-OPRD appear in the family would forward our understanding of the etiological mechanisms of the gCJD.

## Introduction

Prion diseases, also known as transmissible spongiform encephalopathies (TSEs), are a group of rare, fatal neurodegenerative disorders in humans and animals, characterized by the accumulation and aggregation of prions or abnormally folded proteins (1). The abnormally folded proteins PrP^Sc^ have a high number of β-pleated sheets in their posttranslational conformation compared with the typical α-helices seen in the normal form of the protein (PrP^c^). The PrP^Sc^ conformation is partially resistant to proteases and acts as a template for further misfolding of the normal PrP^C^ to abnormal PrP^Sc^. There are three main groups of prion diseases, termed sporadic (Creutzfeldt–Jakob disease [CJD], sporadic fatal insomnia, and variably protease-sensitive prionopathy), genetic (genetic CJD, fatal familial insomnia, and Gerstmann–Straussler–Scheinker syndrome), and acquired (kuru, variant CJD, and iatrogenic CJD). For instance, approximately 85% of CJD cases are sporadic, 10–15% are inherited, and >1% of the cases are acquired (2, 3).

Based on the clinical and pathological features, inherited human prion disease, or genetic prion diseases (gPrDs), are classified as genetic CJD (gCJD) or familial CJD (fCJD), GSS disease, and fatal familial insomnia (FFI). The majority of gCJD have a common feature of rapid progression with a longer survival time; some of them also present as ataxia or Parkinson-like disorders, which have a slower decline over a few to several years (4). The clinical manifestations and neuropathological abnormalities of gPrDs may vary, and the definitive diagnosis of the gCJD requires genetic evidence except for the routine diagnostic elements such as autoimmune encephalitis (5).

The mutation in the prion protein gene (*PRNP*) was identified to contribute to the development of gPrDs. The *PRNP* gene is located at chromosome 20 and encodes prion protein (PrP) with 253 amino acids highly expressed in the central and peripheral nervous systems. The types of mutations in *PRNP* include missense and non-sense mutation, insertions, and deletion (6). Despite that more than 60 *PRNP* variants were identified to be pathogenic to date, five among them, E200K, V210I, V180I, D178N, and P102L account for around 85% of gPrD cases (7).

The G114V *PRNP* variant is a rare subtype in gCJD (8), and only 15 patients from 5 families were reported worldwide. The affected individuals were characterized by an early age of onset, neuropsychiatric symptoms, rapidly progressive dementia, sleep disturbance with predominant pyramidal and extrapyramidal symptoms, and long disease duration (1–5 years) (8–14). Interestingly, a *PRNP* gene variant with one octapeptide repeat deletion (1-OPRD) is highly related to gastric cancer but not prion diseases. In this study, for the first time, we identified a gCJD patient who carries a *PRNP* variant with G114V mutation and 1-OPRD.

## Methods

### Pedigree

**Figure 1:**
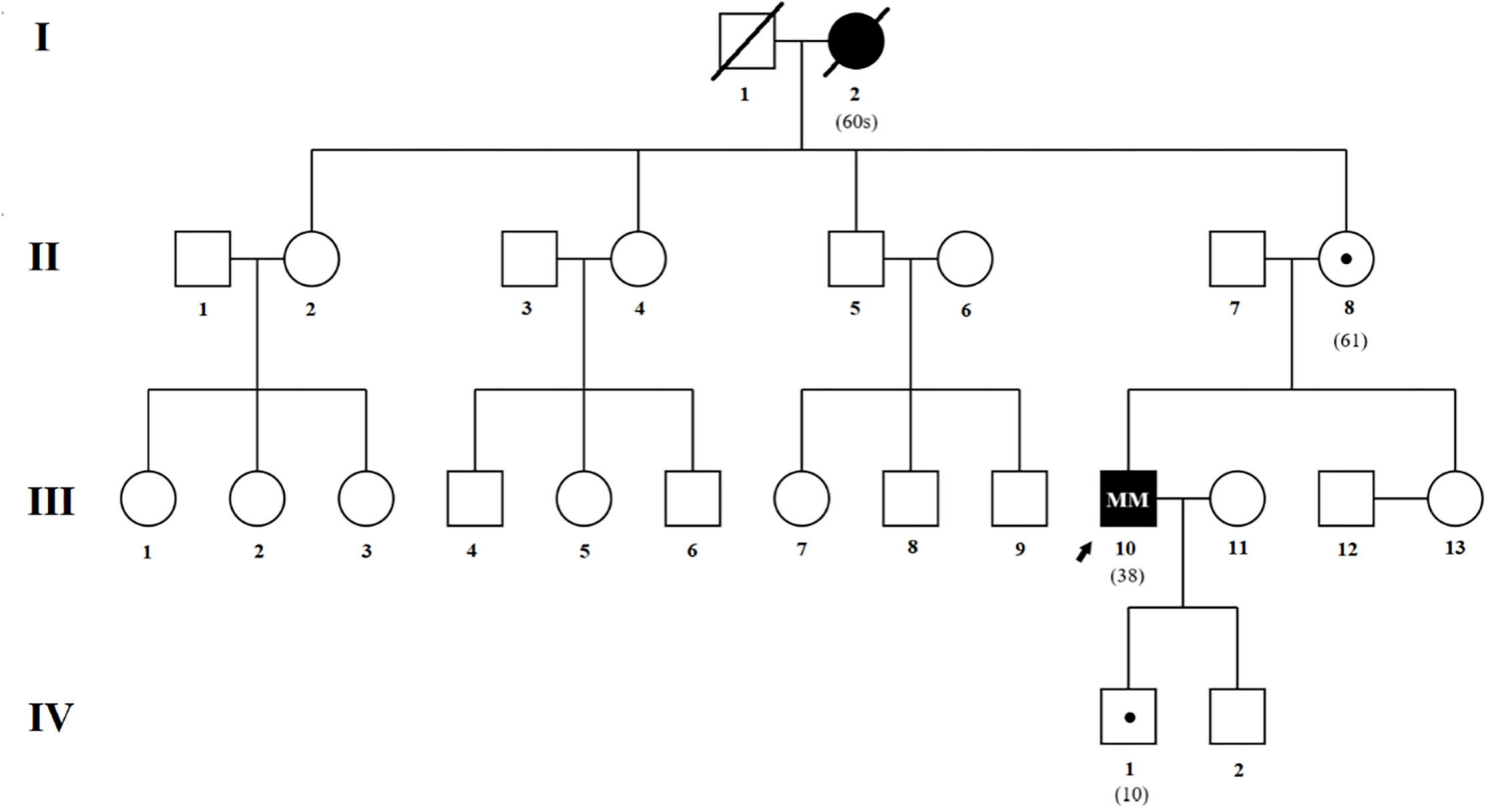
Genealogical tree of the family. Affected patients and asymptomatic carriers are described in the text. Squares indicate males; circles, females; slash marks, deceased; arrow, proband; solid symbols, affected individual; small solid dot with square or circle, asymptomatic carrier.

The pedigree shown in Figure 1 was provided by the proband's mother (II-8).

### Clinical Assessment

The proband (III-10) and one carrier (II-8) underwent systematic neurological and neuropsychological examinations and scoring on the Medical Research Council prion disease rating scale (MRC Scale) (15). This scoring was repeated for the proband and patient II-8 monthly over the telephone in the follow-up studies.

### Genetic Analysis

Genomic DNA was extracted from peripheral blood leucocytes of 3 living members in this pedigree using the DNA Isolation Kit (Bioteke, AU1802). Qualified DNA samples were fragmented into 200~300 bp. The procedure comprises three standard steps: end-repair of fragmented DNA, A-tailing, adapter ligation, and amplification. Hybridization of pooled libraries to the capture probes and removal of non-hybridized library molecules were carried out according to the IDT and xGen Lockdown® Probes (Integrated DNA Technologies). Sample dilution, flow cell loading, and sequencing were performed according to the Illumina specifications. The DNA libraries were sequenced on the HiSeq X10 (Illumina, San Diego, USA) as paired-end 150-bp reads.

### Cerebrospinal Fluid Examination

A lumbar puncture was performed on the proband. Cerebrospinal fluid (CSF) 14-3-3 protein was examined at the National Institute for Viral Disease Control and Prevention, Chinese Center for Disease Control and Prevention.

### Real-Time Quaking-Induced Conversion

CSF and skin RT-QuIC were conducted in the proband at the National Institute for Viral Disease Control and Prevention, Chinese Center for Disease Control and Prevention.

The CSF assay was conducted in a black 96-well, optical-bottomed plate (Nunc, 265301) on a BMG FLUOstar plate reader (BMG LABTECH); 15 μl of each CSF sample was mixed, with 10 μg of recombinant hamster PrP90-231 in a reaction buffer containing 10 mmol/L PBS, 170 mmol/L NaCl, 10 μmol/L thioflavin T (ThT), 10 μmol/L EDTA, and 0.002% SDS in final. The final reaction volume was 100 μl. Each tested sample was quadruplicated. Each reaction contained blank (reaction buffer), negative (2 μl 10% brain homogenate from normal hamster), and positive (2 μl 10% brain homogenate from scrapie agent 263K-infected hamster) controls. The working conditions were as follows: temperature, 55 °C; shaking speed, 700 rpm; shaking/incubation time, 60/60 s; total reaction time, 60 h. ThT fluorescence (450 nm excitation and 480 nm emission) was automatically measured every 45 min as relative fluorescence units (rfu). The cutoff value was set as the average value of the negative controls plus 10 times SD. The sample was considered positive when two or more parallel wells revealed positive reactive curves.

The sites for skin biopsies were behind the right ear. After disinfection with 75% alcohol, the proband received local anesthesia with a subcutaneous injection of 2% lidocaine hydrochloride. A small piece of skin with a size of about 2 × 1 cm^2^ was taken with a scalpel by a neurosurgeon. The biopsy skin specimen covered the epidermis, dermis, and adipose tissues; 2% (w/v) of skin homogenate was prepared in lysis buffer (100 mM NaCl, 10 mM EDTA, 0.5% Nonidet P-40, 0.5% sodium deoxycholate, 10 mM Tris, pH 7.5). RT-QuIC reaction contained 10 μg of rHaPrP90-231, 1X PBS, 170 mM NaCl, 1 mM EDTA, 0.01 mM ThT, 0.001% SDS, together with 15 μl CSF samples or 2 μl 10^−2^ to 10^−4^ diluted skin homogenates in a final volume of 100 μl. Each sample was assayed in triplicated or quadruplicated. The assay was conducted in a black 96-well, optical-bottomed plate (Nunc, 265301) on a BMG FLUOstar plate reader (BMG LABTECH). The working conditions were optimized as follows: temperature, 55°C; vibration speed, 700 rpm; vibration/incubation time, 60/60 s; total reaction time, 60 h. ThT fluorescence (excitation wavelength, 450 nm; emission wavelength, 480 nm). Each reaction was automatically measured every 45 min and expressed as relative fluorescence units (rfu). The cutoff value was set as the mean value of the negative controls plus 10 times the standard deviation. A sample was considered to be positive when ≥2 wells revealed positive reaction curves. The positive control was 10^−5^ diluted the brain homogenate of the scrapie agent 263K-infected hamster, while the negative control was 10^−5^ diluted the brain homogenate of the normal hamster.

### Ethical Approval and Consent to Participate

All patients were informed about the purpose of the study and given written consent. The study was approved by the Ethics Committee of the Beijing Tongren Hospital.

## Results

### Case Report

A 36-year-old Han-Chinese right-handed man (patient III-10, the proband) developed anxiety, depression, sleep disorder, and tremor in his hands after a panic attack but without known medical history. His cognitive state declined, and he was unable to perform job duties due to memory loss. At 11 months after onset, the patient became withdrawn, and more deterioration of his cognitive function was observed, indicated by the difficulty in calculating, being lost at home, and frequently forgetting the names of acquaintances. Besides, the tremors spread to bilateral limbs, which led to difficulty in cake decoration (the patient's profession). After 16 months of onset, he was diagnosed with depression and anxiety and was prescribed sertraline, fluphenazine, and piracetam. At 17 months after onset, he was scored 20 of 30 on the Chinese Mini-Mental Status Examination (MMSE), and 15 of 30 on the Montreal Cognitive Assessment (MoCA Beijing Version). The Hamilton's Depression Scale was 7. At 19 months after onset, he developed hallucinations, which made him see his sons as enemies, and occasionally, he protected himself *via* aggressive behaviors.

In the clinic, the diagnosis of possible behavioral variant Frontotemporal Dementia (bvFTD) was considered because of the abnormal neuropsychological profile, such as early apathy and executive/gene deficits with relative sparing of memory and visuospatial functions.

A thorough neurological examination 20 months after the onset revealed a total deterioration of the cognitive state, dysarthria, slight hypermyotonia, and deep tendon hyperreflexias in the bilateral limbs, and the patient presented cogwheel-like rigidity. He had difficulty finishing the finger-to-nose test and heel-knee-tibia test due to tremors in his limbs. The patient was unsteady when walking on a straight line. No involuntary movement was observed. He scored 15 of 30 on the Chinese MMSE, and 9 of 30 on the MoCA Beijing Version. The Hamilton's Depression Scale was 7.

**Figure 2:**
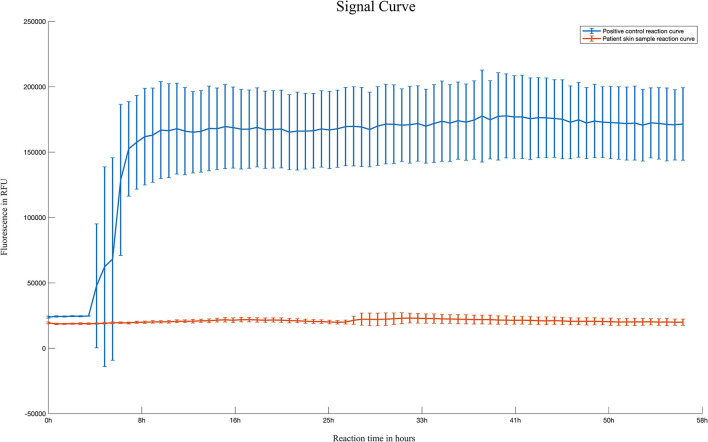
Reaction curve of skin RT-QuIC of the patient. Reaction curves of skin RT-QuIC of the patient. Four replicate reactions of patient skin samples and four replicate reactions of the positive control (hamster scrapie 263K strain brain smear with a 10^−7^ dilution) were used in the RT-QuIC. Each line represents the reaction curve of positive control and patient sample, respectively. The means with standard deviations of those averages are shown as a function of RT-QuIC reaction time. The reaction curve did not show the peak of fluorescence in the patient sample (bottom), indicating a negative result of RT-QuIC. X-axis, hours post-reaction; Y-axis, fluorescence values.

To our surprise, the brain MRI demonstrated abnormal intensities in the bilateral caudate nucleus, putamen, and cerebral cortex. A series of laboratory examinations were performed for the rapidly progressive early-onset dementia. The autoimmune screening and tumor marker identification were shown to be unremarkable or negative. An extensive panel for paraneoplastic antibodies including Amphiphysin, CV2, PNMA2 (Ma2/Ta), Ri, Yo, Hu, titin, SOX1, recoverin, zic4, GAD65, and Tr (DNER) were tested, and all were negative. Serology and cerebrospinal fluid (CSF) tests for HIV, cryptococcus, syphilis, tuberculosis, bacteria, fungus, and virus showed no evidence of inflammation. In addition, CSF 14-3-3 protein was tested to be negative. RT-QuIC tests of skin and CSF were also negative (Figure 2).

**Figure 3:**
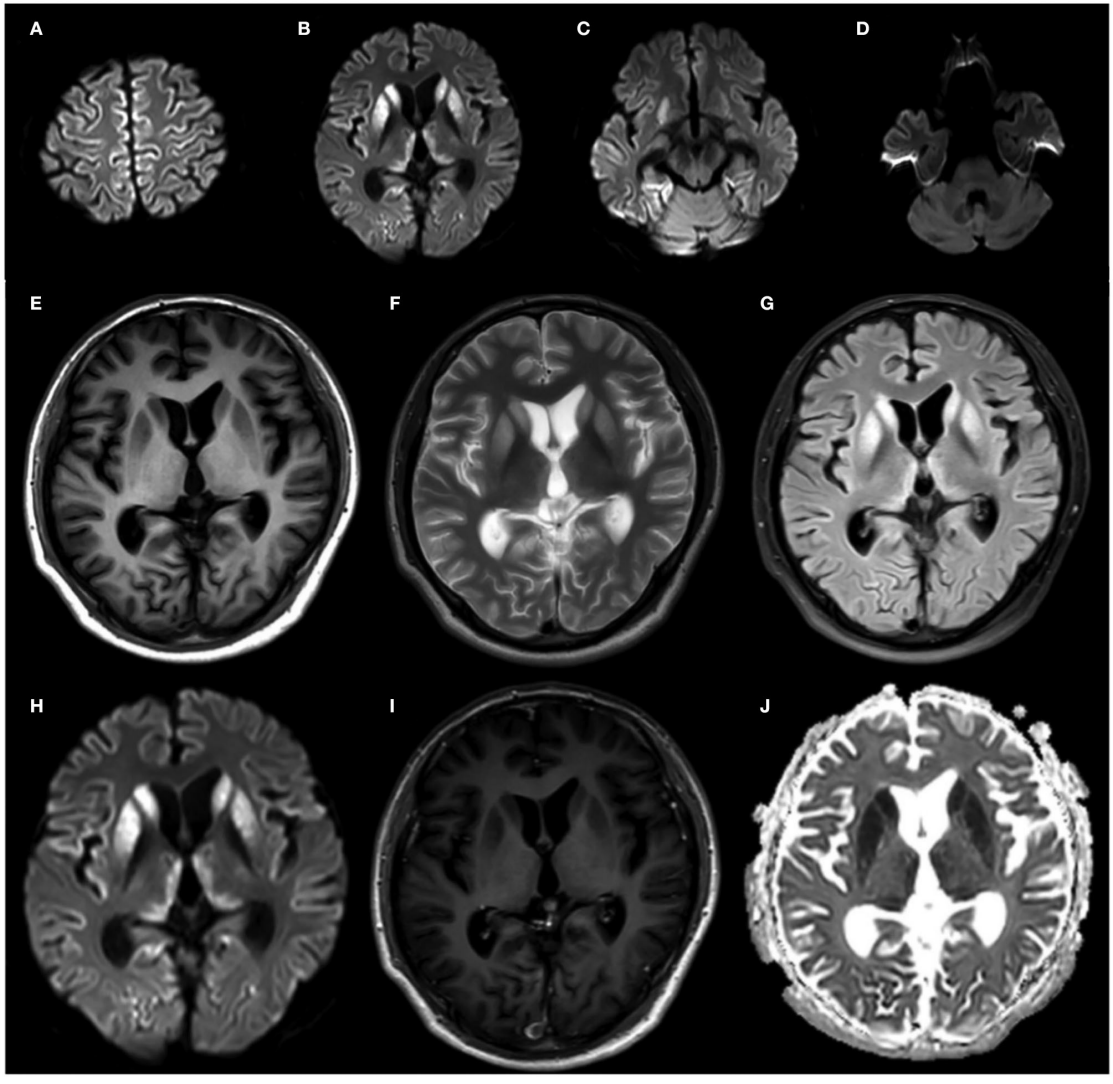
Brain MRI of the proband at 20 months after onset. **(A–D)** DWI sequences displayed restricted diffusion in the bilateral frontal and parietal cortex. **(E–J)** Bilateral basal ganglia hyperintensities, with slight bilateral pulvinar and dorsomedial thalamus hyperintensities on FLAIR **(I)** and DWI **(J)** sequences.

**Figure 4:**
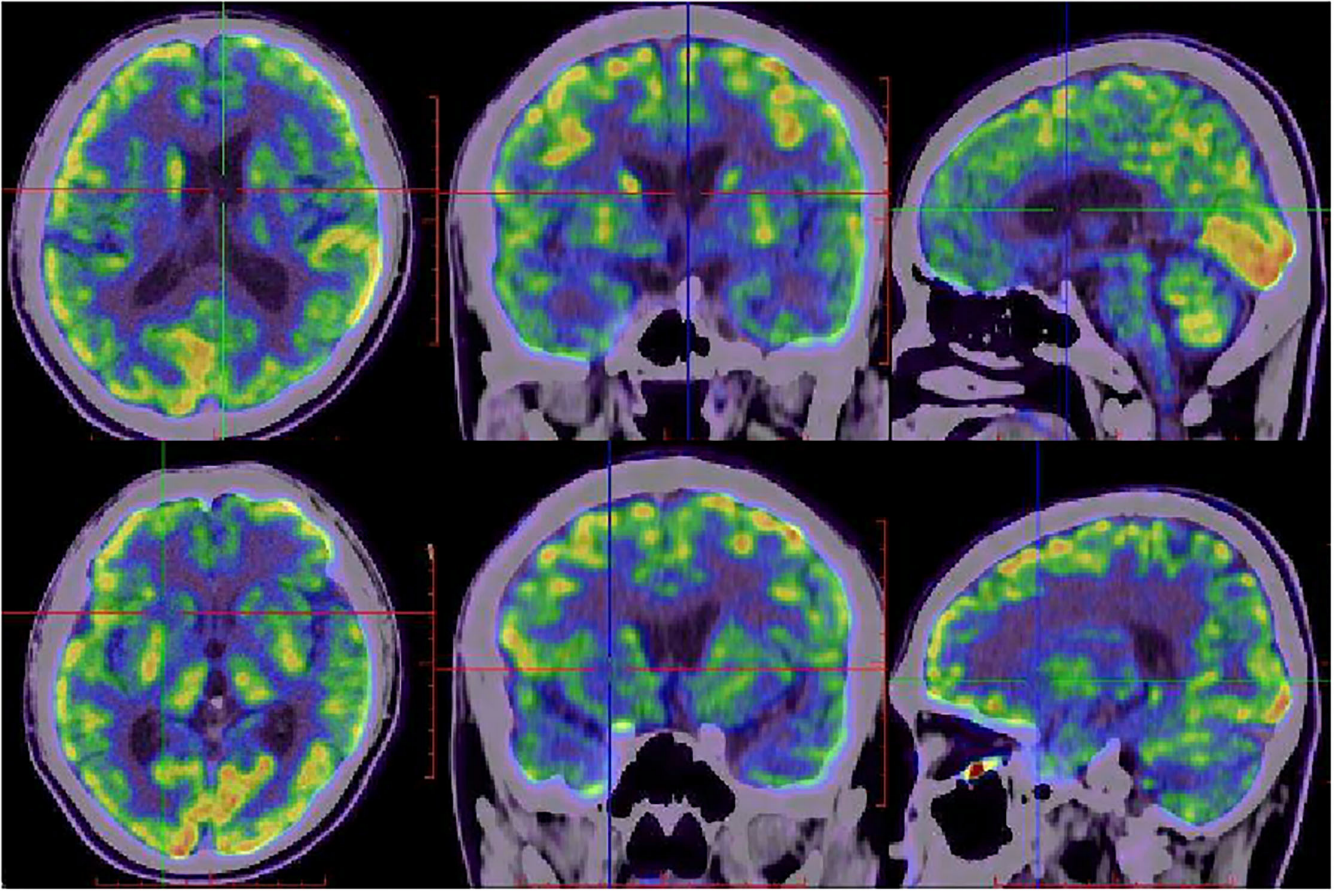
FDG-PET images of the proband at 20 months after onset. FDG-PET showed hypometabolism in bilateral frontal, parietal, temporal lobes, bilateral caudate nucleus, putamen, and thalamus, with right lateralized basal ganglia hypometabolism.

Diffusion-weighted imaging (DWI) sequences displayed restricted diffusion in the bilateral frontal and parietal cortex (Figures 3A–D). DWI hyperintensities were revealed in the bilateral basal ganglia, bilateral pulvinar, and dorsomedial thalamus (Figures 3E–J). Fluorodeoxyglucose PET (FDG-PET) exhibited hypometabolism in the bilateral cerebral cortex and right basal ganglia (Figure 4).

An empiric course of pulse IV gamma globulin was tried without notable improvement.

At 22 months after onset, the hypermyotonia of the patient in the bilateral limbs became more obvious. He had occasional urinary incontinence, and his ability to study was relatively reserved when he scored 21 of 30 on the Chinese MMSE. The MRC Scale score was 13 of 20. The Hamilton's Depression Scale was 6. EEG showed diffuse slow waves. MRI scanning indicated no obvious change compared to images 2 months earlier.

A follow-up study with the MRC Scale revealed gradually developed aphasia, gait disorder, and fecal incontinence 30 months after onset. The patient is still alive, 4 years after the onset, while the MRC Scale score was 2 of 20, and his swallowing function and mobility were still preserved (Table 1).

**Table 1:** The MRC Scale of the proband.

**MRC scale**	**Duration (months)**									
	**20**	**22**	**28**	**31**	**34**	**37**	**40**	**43**	**46**	**49**
Bowel function	1	1	1	0	0	0	0	0	0	0
Bladder function	1	1	1	0	0	0	0	0	0	0
Toilet use	1	1	1	1	1	1	1	0	0	0
Bathing	1	1	1	1	1	0	0	0	0	0
Feeding	2	1	1	1	1	1	1	1	1	1
Transfers and mobility	1	1	1	1	1	1	1	1	1	0
Stairs	1	1	1	1	1	1	0	0	0	0
Best verbal response	3	2	2	1	1	0	0	0	0	0
Memory and orientation to surroundings	2	1	1	1	1	1	0	0	0	0
Judgement and problem solving	1	1	1	1	1	0	0	0	0	0
Use of tools	1	1	1	1	1	0	0	0	0	0
Total	15	13	13	9	9	6	3	2	2	1

**Figure 5:**
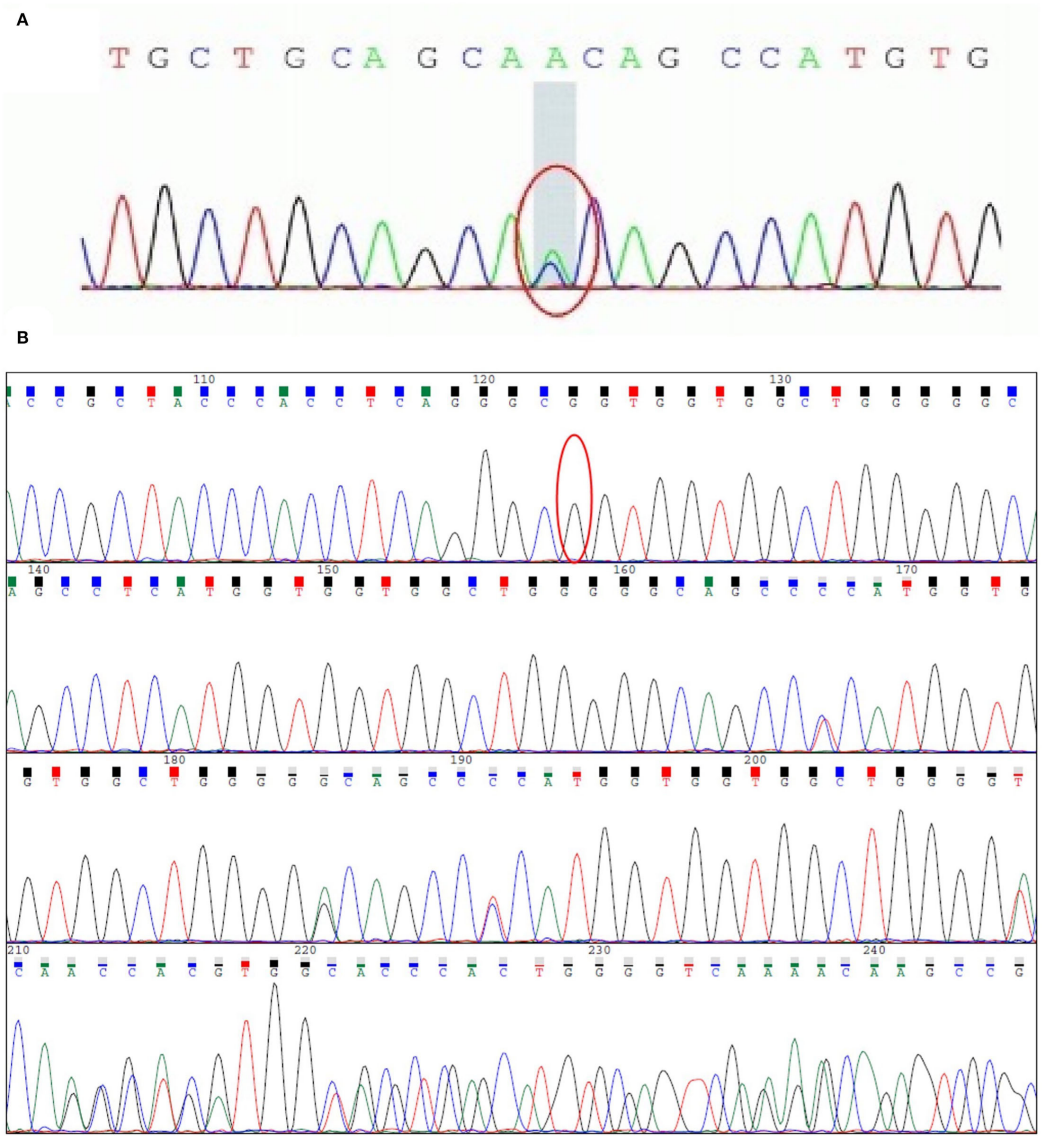
Sequencing analyses in the *PRNP* gene from patient III-10. **(A)** c.341G>T (p.G114V) variant of the PRNP gene was detected. The mutation leads to the alteration of amino acid from glycine-to-valine. This alteration will lead to prion disease. **(B)** c.204_227 delTCATGGTGGTGGCTGGGGGCAGCC (1-OPRD) variant was detected. PRNP gene comprises two exons with the entire open reading frame (ORF) of 762 bp which is contained with exon 2. Nuclides between PRNP codon 51-91 into N-terminal are 5 groups of 24 base pair (bp) repeats, a deletion of the repeat TCATGGTGGTGGCTGGGGGCAGCC was found at the end of the PRNP gene (bottom). This mutation can be found in a minority of people and its pathogenicity has not yet been determined.

The clinical features indicated that the patient might have prion disease. To determine the etiology of the disease, we extracted genome DNA from peripheral blood leucocytes of the patient and performed a direct DNA sequencing of the *PRNP* coding sequence. Unexpectedly, a rare mutation of G114V and 1-OPRD of the PrP in the patient was identified (Figure 5). Given that only a few patients with gCJD were reported to carry the G114V *PRNP* variant, we suspected that the mutation in the patient might be inherited from his parents, and we, thus, enrolled his immediate family members in this study for a genetic investigation.

Although the medical record was not available, the maternal grandmother (patient I-2) of the proband was found to have progressive dementia in her 60s, which was within 1 year before her death, and subsequently developed a tremor in her last few months. The proband's mother (carrier II-8) received examination when she was 61 years old, but no neuropsychiatric symptoms were observed. She scored 29 of 30 on the Chinese MMSE and 21 of 30 on the MoCA (Beijing Version). Although the EEG showed slow waves in the left temporal lobe, both cranial MRI (including DWI) and FDG-PET were unremarkable. A follow-up study revealed that she suffered an acute cerebral infarction in the callosum 18 months after the first examination. No cortical ribbon was found by DWI to date (Table 2). Whereas DNA sequencing revealed a G114V mutation and 1-OPRD of PrP in this individual. The elder son of the proband (carrier IV-1) received DNA sequencing at age 10, and G114V mutation and 1-OPRD were also found, although he did not show any clinical symptoms. No further examination was performed due to his age. No autopsy or biopsy were performed on any of the patients.

**Table 2:** Summary of clinical features in affected family members.

**Case no**.	**Sex**	**Age at onset**	**Illness of** **duration, y**	**Neuropsychiatric symptom**	**Dementia**	**Corticospinal** **signs**	**Extrapyramidal signs**	**Ataxia**	**Myoclonus**
III-10	Male	36	4	+	+	-	+	+	+
I-2	Female	60s	<1	NA	+	NA	NA	NA	+

*(+), present; (-), absent; NA, not available*.

### Genetic Analysis

Direct sequencing of the *PRNP* coding sequence disclosed a heterozygous missense mutation (Figure 5A) at the second position of codon 114, leading to a GGT-to-GTT substitution and a glycine-to-valine change in the PrP (G114V). 1-OPRD was also identified by the sequencing (Figure 5B). Both the mutation and deletion were found in 3 family members (Figure 1), including patients III-10, and asymptomatic carriers II-8 and IV-1.

### Neuropsychological Assessment

At 20 months and 22 months after onset, a series of cognitive investigations were carried out to evaluate the proband's cognitive state (Table 3). At 20 months after onset, he scored 15 of 30 on the Chinese MMSE, 9 of 30 on the MoCA Beijing Version, 0 of 3 on the Clock Drawing Test (CDT), 38 of 40 on the Boston Naming Test (BNT), and 3 of 10 on the Copying test. The Hamilton's Depression Scale was 7. The Digit span test (DST) showed 7 in order and 3 in inverted order. The symbol digit modalities test (SDMT) score was 0 of 110. The score of the Rey auditory verbal learning test (RAVLT) exhibited that the ability to learn and recall was significantly reduced. The proband could not finish both the Trail making test (TMT)-A and B. The activity of daily living (ADL) scale and instrumental activities of daily living (IADL) scale displayed 15 of 40 and 32 of 40, respectively. Besides, he got 6 on the clinical dementia rating scale sum of boxes (CDR-SB). The MRC Prion Disease Rating Scale (MRC Scale) was scored 15 of 20.

**Table 3:** Summary of cognitive abilities test in examined family members.

	**III-10**	**III-10**	**III-10**	**II-8**
	**(17 months)**	**(20 months)**	**(22 months)**	
MMSE	20	15	21	29
MoCA	15	9	17	21
CDR-SB	NA	6	6	0.5
BNT (20)	NA	19	20	17
CDT	NA	0	1	3
TMT - A, s	NA	NA	240	40
TMT - B, s	NA	NA	NA	NA
DST - in order	NA	7	6	8
DST - inverted order	NA	3	3	3
SDMT	NA	0	0	24
ADL	NA	15	18	10
IADL	NA	32	38	11
Copying	NA	3	3	8
Calculation	NA	3	4	9
MRC Scale	NA	15	13	20

At 22 months after onset, he scored 21 of 30 on the Chinese MMSE, 17 of 30 on the MoCA Beijing Version, 20 of 20 on BNT, 1 of 3 on CDT, and 3 of 10 on the Copying test. DST showed 6 in order and 3 in inverted order. The Hamilton's Depression Scale was 7. SDMT score was 0 of 110. He still got 6 on CDR-SB. The score of RAVLT showed that the patient's ability of learning and recall was increasingly damaged. The TMT-A was finished correctly in 240 s while the TMT-B was not finished. ADL displayed 18 of 40 in PSMS and 38 of 40 in IADL. The score of the MRC Scale was 13 of 20.

## Discussion

In this report, we have described a rare G114V mutation and 1-OPRD in PrP in a Chinese family comprising two affected individuals (III-10 and I-2) and two carriers (II-8 and IV-1) (Figure 1). II-8 and III-10 were alive at examinations. To the best of our knowledge, our report is the first description of a CJD case with both G114V mutation and 1-OPRD *PRNP* variant.

The proband developed anxiety, depression and progressive dementia, myoclonus, pyramidal, and extrapyramidal syndrome in his late 30s. The DWI sequences in cranial MRI of the patient displayed restricted diffusion in the cortical ribbon, bilateral basal ganglia, bilateral pulvinar, and dorsomedial thalamus hyperintensities. FDG-PET exhibited hypometabolism in the cerebral cortex and the right basal ganglia. The patient was suspected of having CJD while the 14-3-3 protein in CSF was not detected and the RT-QuIC for PrP^Sc^ was negative. Finally, the genetic analysis showed the G114V *PRNP* variant with 1-OPRD.

G114V is a rare mutation linked to the early age of onset, ranging from 18 to 45 years, and long disease duration (1 to 5 years). Since 2005, only 15 patients with G114V-associated genetic prion diseases were identified (the major clinical characteristics are summarized in Table 4), and the appearance of G114V mutation seems to have no obvious racial difference. Among the 15 cases, progressive dementia appeared in all cases (100%), extrapyramidal syndromes were reported in 12 cases (80%), and neuropsychiatric symptoms were described in 10 cases (66%). Myoclonus was mentioned in 9 cases (60%) and pyramidal syndromes were noted in 8 cases (53%). Additionally, mild cerebellar signs were reported in a Uruguayan family, and aphasia was described in an American-born Polish descent family (9, 12). Almost all of the patients had neuropsychiatric symptoms and progressive dementia at the onset. In all 15 cases, progressive dementia gradually appeared and lasted the entire clinical course.

**Table 4:** Features of the 15 reported cases with PrP G114V mutation.

**Case no**.	**Gender**	**Age at onset (y)**	**Familiy history**	**Symptoms at onset**	**Clinical symptoms**								**CSF 14-3-3**	**EEG (PSWC)**	**MRI**	**FDG-PET**	**Genetic analysis**	**Duration of illness (y)**
					**Progressive dementia**	**Personality behavioral changes**	**Myoclous**	**Visual hallucination**	**Cerebellar disturbance**	**Pyramidal dysfunction**	**Extrapyramidal dysfunction**	**Akinetic mutism**						
(2005, Rodriguez) Case III-14	Male	27	Positive	Behavior Change	+	+	+	+	-	+	+	+	NA	-	NA	NA	PRNP G114V mutation	2
(2005, Rodriguez) Case III-16	Male	18	Positive	Mood and behavior change	+	+	+	-	-	NA	+	+	NA	-	NA	NA	NA	1
(2005, Rodriguez) Case III-19	Female	22	Positive	behavioral changes, loss of memory, hallucinations	+	+	+	-	+	+	+	-	NA	-	Moderate diffuse encephalic atrophy	NA	PRNP G114V mutation	2
(2005, Rodriguez) Case IV-2	Female	22	Positive	Personality changes	+	+	+	-	+	+	+	+	NA	-	Diffuse cerebral atrophy	NA	PRNP G114V mutation	4
(2005, Rodriguez) Case II-1	Male	28	Positive	Psychiatric symptoms	+	+	NA	NA	NA	NA	NA	NA	NA	NA	NA	NA	NA	2
(2009, Liu) Case III-4	Female	45	Positive	Psychiatric symptoms, progressive dementia	+	+	-	+	-	+	+	-	-	-	Moderate diffuse cerebral atrophy, especially hippocampi bilaterally. DWI hyperintensities in the bilateral caudate nuclei and the putamen, and slight ribbon hyperintensities in the insular cortices.	NA	PRNP G114V mutation	NA
(2009, Liu) Case III-2	Male	45	Positive	Rapidly progressive dementia	+	+	-	-	-	-	+	-	NA	-	Moderate diffuse cerebral atrophy	NA	NA	2
(2009, Liu) Case III-3	Female	35	Positive	Psychiatric symptoms	+	+	-	-	-	-	-	-	NA	NA	NA	NA	NA	2.5
(2009, Liu) Case IV-2	Male	32	Positive	Rapidly progressive dementia	+	-	-	-	-	+	+	-	NA	NA	Normal	NA	PRNP G114V mutation	NA
(2018, Cali) IV-A	Male	24	Positive	Psychiatric symptoms, progressive dementia	+	+	+	+	+	+	+	-	-	-	Restricted diffusion throughout the cortical ribbon, and right, greater than left, cortical atrophy	Decreased but symmetric cortical metabolism, more pronounced in the posterior parietal, temporal, and occipital regions, thalamic uptake was similar to the cortex, and a relative increase in metabolic activity in the basal ganglia.	PRNP G114V mutation	5
(2018, Cali) III-B	Male	32	Positive	Rapidly progressive dementia	+	-	+	+	+	-	+	+	NA	NA	Diffuse cerebral atrophy, markedly out of proportion for age (report only)	NA	NA	2
(2018, Margolesky) Case	Female	20	Negative	Rapidly progressive dementia, extrapyramidal dysfunction	+	-	+	-	-	+	+	-	NA	-	Abnormal signal in the cortex diffusely and striatum bilaterally	NA	PRNP G114V mutation	NA
(2018, Cousyn) Case	Male	36	Negative	Rapidly progressive dementia	+	-	+	-	-	+	+	-	-	-	Hyperintensities of parieto-occipital cortex	NA	PRNP G114V mutation	4
III-10	Male	36	Positive	Psychiatric symptoms	+	+	+	+	+	-	+	+	-	-	Restricted diffusion in cortical ribbon, bilateral basal ganglia hyperintensities, with bilateral pulvinar and dorsomedial thalamus hyperintensities	Hypometabolism in right basal ganglia	PRNP G114V mutation	5
I-2	Female	60s	Positive	Rapidly progressive dementia	+	NA	+	NA	NA	NA	NA	NA	NA	NA	NA	NA	NA	<1

The 14-3-3 protein is one of the most common laboratory markers in the diagnosis of CJD for its high sensitivity and specificity. But Muayqil et al. (16) indicate that it can be positive in different neurological diseases, including encephalitis and acute stroke. The 14-3-3 protein was shown to be negative in the proband CSF. Interestingly, CSF 14-3-3 is negative in all reported G114V patients (8–14), which suggests that 14-3-3 protein is not a specific index for this phenotype.

RT-QuIC for PrP^Sc^ in skin and CSF were also negative in the proband, which was not available in other cohorts. These results were first reported in G114V-gCJD. The diagnostic value of RT-QuIC in the detection of sCJD from CSF samples has been demonstrated previously (17). Orrù et al. also reported that the RT-QuIC-based method detects CJD patients with an overall sensitivity and specificity of 100% (18). Besides, according to Xiao et al. skin specimen was ideal for the RT-QuIC test in Chinese patients (19). The RT-QuIC assay was negative in many other gCJDs, such as V180I, V210I mutation (20, 21).

A detailed neuropsychological investigation revealed that the proband was impaired in language and recall and developed time disorientation, attention deficit disorder, dysexecutive syndrome, and visuospatial dysfunction, which is consistent with previous reports. For example, Cousyn et al. reported dysexecutive syndrome and impaired episodic memory with spatial disorientation in their cases (14). A major axis of the frontoparietal dysfunction was also reported in a study by Caine et al. which includes patients with inherited, acquired, and sporadic CJD, suggesting characteristic cognitive features in prominent executive impairment, parietal dysfunction, a largely expressive dysphasia with reduced motor speed, and it strongly correlated with volume reduction in the frontal and parietal gray matter revealed by MRI (22).

The MRC Scale examination assesses domains of cognitive function, speech, mobility, personal care/feeding, and continence, according to their relative importance documented by the carer's interviews, which can describe the disease progression of a patient. The 20-point functionally oriented scale used in this study is advantageous over single scales for its simplicity of administration and the ability to capture the rapid changes that can characterize the disease. The scores of the MRC Scale for the patient from 20 months to 49 months after disease onset are summarized in Table 1, which showed that memory and orientation to surroundings were relatively reserved through long disease duration.

The DWI imaging revealed hyperintensities in the bilateral basal ganglia, pulvinar, and dorsomedial thalamus of the patient. However, hyperintensities in the cortex were restricted to the bilateral frontal and parietal cortex. A similar phenomenon was noted in previously reported one Chinese and two American families carrying the G114V mutation. Compared to another type of gCJD with E200K mutant or sCJD, the ribbon sign is less evident in the cortex in all the G114V patients.

Recently, FDG-PET has been suggested to be used in the diagnosis of CJD, but literature about utilizing FDG-PET in gCJDs is limited (23). In our report, FDG-PET exhibited hypometabolism in the right basal ganglia and cortex of the proband, which is consistent with a previous report that a relative increase in metabolic activity in the basal ganglia as revealed by FDG-PET in a G114V case (12). Interestingly, the basal ganglia and thalamus were unaffected in the context of metabolism of sCJD, as reported previously (24). Thus, measuring the metabolism activity in basal ganglia may help diagnose gCJD.

The EEG of the proband showed diffuse slow waves without typical periodic sharp wave complexes, which are similar to previous reports.

Amino acid 114 is located within a potential membrane-spanning domain of PrP^C^, which not only belongs to a highly conserved palindromic sequence but also to an amyloidogenic region that is essential for the conversion of PrP (25). G114V has been demonstrated to associate with some pathways, including oxidative phosphorylation, regulation of actin cytoskeleton, MAPK signaling and proteasome, axon guidance, gap junction, and purine metabolism (26). Cali et al. indicated that the conformation of PrP^Sc^ linked to the PRNP-G114V variant might be the principal barrier to transmission (12).

In addition to the G114V mutation, we also detected 1-OPRD in the PrP in the gCJD patient, which has never been reported in the prion diseases. The PRNP gene comprises two exons with the entire open reading frame (ORF) of 762 bp which is contained with exon 2. Nuclides between PRNP codon 51-91 into N-terminal are 5 groups of 24 base pair (bp) repeats, which are also called octapeptide repeats region (OPRP). RNP gene are transmitted in an autosomal dominant manner and include point mutations (such as G114V, E200K, and T188K), insertion of one-nine 24 bp extra repeats or two 24 bp repeats deletion between PRNP codon 51-91 (27, 28). Insertional mutations of one or more extra octapeptide (from 2 to 12 OR insertions) are associated with CJD (4, 29). Two-octapeptide repeat region (2-OPRD) deletions were reported in two unrelated patients and were considered to be pathogenic (6, 30). The two 2-OPRD cases were characterized by late age of onset (86 and 62 years old) and rapidly progressive dementia. 1-OPRD *PRNP* variant has also been found in healthy individuals with a frequency of less than 1% (31). Although the biological functions of 1-OPRD largely remain unclear, one report suggested that single octapeptide deletion selectively reduces the expression level of pathogenic N-terminal mutants. Besides, 1-OPRD also induces the release of a pathogenic PrP mutant that the mutation occurred within the internal hydrophobic domain from the cell surface (32). 1-OPRD also exists in HeLa cells and several gastric cancer cell lines. Around 66.7% variant frequency in gastric cancer cell lines and 8.9% in gastric cancer tissues were reported, which was significantly higher than that in the normal population (less than 1%) (33, 34). Our report first described 1-OPRD in a familial prion disease, which suggests that 1-OPRD plays a role in the pathogenesis of prion disease.

In summary, we reported a rare pedigree with PrP G114V mutation combined with 1-OPRD, the first report in human gCJD. The proband and his mother and his elder son carry the same mutations. The proband displayed symptoms at the age of 36, but his mother did not develop prion-like disorders in her 60s. The phenomenon would help to forward our understanding of the etiology of gCJD and warrants future study on its pathologic mechanisms.

## Data Availability Statement

The datasets presented in this article are not readily available because of ethical and privacy restrictions. Requests to access the datasets should be directed to the corresponding author/s.

## Ethics Statement

The studies involving human participants were reviewed and approved by Ethics Committee of Beijing Tongren Hospital. The patients/participants provided their written informed consent to participate in this study.

## Author Contributions

XL and YX drafted the initial manuscript and reviewed the literature. XL, YX, and ZZ were in charge of the patients' follow up and cognitive assessments. KX, XC, QS, and XD performed the Skin RT-Quic. JY and HG provide part of imaging. JW and YG revised the manuscript and in charge of all the clinical data analysis, gene analysis, and imaging. All authors contributed to the article and approved the submitted version.

## Funding

This work was supported by the National Natural Science Foundation of China grant (81771313 and 81301032), Open Project of State Key Laboratory of Infectious Disease Prevention and Control (2020SKLID311).

## Conflict of Interest

The authors declare that the research was conducted in the absence of any commercial or financial relationships that could be construed as a potential conflict of interest. The handling editor LW declared a shared parent affiliation with the author(s) XL, YX, ZZ, XC, JY, and YG at the time of review.

## Publisher's Note

All claims expressed in this article are solely those of the authors and do not necessarily represent those of their affiliated organizations, or those of the publisher, the editors and the reviewers. Any product that may be evaluated in this article, or claim that may be made by its manufacturer, is not guaranteed or endorsed by the publisher.
